# MdbZIP44–MdCPRF2-like–*Mdα-GP2* regulate starch and sugar metabolism in apple under nitrogen supply

**DOI:** 10.1093/hr/uhae072

**Published:** 2024-03-15

**Authors:** Xuejing Cao, Zhigang Guo, Ping Wang, Shixiong Lu, Wenfang Li, Zonghuan Ma, Juan Mao, Baihong Chen

**Affiliations:** College of Horticulture, Gansu Agricultural University, Lanzhou 730070, China; College of Bioengineering and Biotechnology, Tianshui Normal University, Tianshui 741000, China; College of Horticulture, Gansu Agricultural University, Lanzhou 730070, China; College of Horticulture, Gansu Agricultural University, Lanzhou 730070, China; College of Horticulture, Gansu Agricultural University, Lanzhou 730070, China; College of Horticulture, Gansu Agricultural University, Lanzhou 730070, China; College of Horticulture, Gansu Agricultural University, Lanzhou 730070, China; College of Horticulture, Gansu Agricultural University, Lanzhou 730070, China

## Abstract

Nitrogen (N) is regarded as an essential macronutrient and is tightly associated with carbon (C) metabolism in plants. The transcriptome data obtained from this study showed that the expression level of the apple basic leucine zipper (bZIP) transcription factor (TF) *MdbZIP44* was up-regulated in ‘Oregon Spur Delicious’ (*Malus domestica* Borkh.) apple fruits under nitrogen supply. MdbZIP44 bound to the promoter of *Mdα-GP2* gene and inhibited its expression, thereby promoting starch accumulation and decreasing glucose content in apple and tomato fruits. Besides, overexpression of *MdbZIP44* promoted sucrose accumulation by regulating the activities of sucrose metabolism-related enzymes and the expression of sugar metabolism-related genes in apple callus and tomato fruits. Furthermore, biochemical assays indicated that MdbZIP44 directly interacted with MdCPRF2-like, another bZIP gene in apple. Meanwhile, this study found that MdCPRF2-like, along with the MdbZIP44 and MdCPRF2-like complex, could activate the expression of *Mdα-GP2*, respectively. In conclusion, this study provides a new reference for potential mechanisms underlying that MdbZIP44–MdCPRF2-like–*Mdα-GP2* regulates starch and sugar metabolism under nitrogen supply.

## Introduction

Apple is an essential fruit worldwide with significant nutritional and economic value. As the leading apple producer, China faces a significant challenge in its primary cultivation regions due to nitrogen deficiency [1]. Nitrogen is involved in numerous physiological and biochemical processes, including the synthesis of nucleic acids, proteins, chlorophyll, phospholipids, vitamins, hormones, and alkaloids, playing a vital role in plant development and fruit yield and quality formation [2, 3]. Insufficient nitrogen supply will lead to weak plant growth and reduced yield, while excessive nitrogen supply results in higher production costs and environmental pollution [4]. Meanwhile, fruit quality is another crucial economic trait of horticultural crops, highly influenced by soluble sugar. Soluble sugar has a significant impact on the sweetness, texture, and preservation ability of the fruit, thus affecting the taste preference of consumers. Therefore, understanding the relationship between carbohydrates and different nitrogen levels is beneficial for improving the quality and yield of horticultural crops. Recent research indicates that bZIP transcription factors (TFs) regulate carbohydrate accumulation and amino acid metabolism in pear [5]. However, the role of bZIP TFs in apple carbon and nitrogen metabolism remains largely unknown.

bZIP TFs are named based on their conserved domain with 60–80 amino acids. This domain includes an N-terminal region composed of 18 basic amino acids containing a nuclear localization signal (NLS) binding to a specific DNA sequence motif, N-x7-R/K. The C-terminus is a leucine zipper region that often interacts to form homo- or heterodimers in an α-helical form due to its unique amino acid composition [6]. For example, the bZIP18 and bZIP34 TFs in *Arabidopsis* pollen can interact to form heterodimers regulating lipid metabolism pathways and affecting pollen wall synthesis [7]. *AtbZIP10* and *AtbZIP53* form a heterodimer that specifically binds to the promoter of proline dehydrogenase, thereby activating proline dehydrogenase expression under hypotonic stress [8]. bZIP TFs usually preferentially bind to ACGT *cis*-acting elements, including G-box (CACGTG), C-box (GACGTC), A-box (TACGTA), and ABRE (CCACGTGG). Studies have shown that several bZIP TFs from the S1 subgroups (bZIP1, 2, 11, 44, and 53) and C subgroups (bZIP9, 10, 25, and 63) are involved in sugar signaling in plants [8, 9]. Sagor *et al.* found that the *SlbZIP1* could increase sugar content in tomato fruits by displaying SIRT (sucrose-induced translation inhibition), without any growth retardation for the tomato plants [10]. By using gene editing, the *FvebZIPs1.1* gene created seven new alleles in wild strawberries, and the homozygous T1 mutant had a sugar content that was 33.9% to 83.6% higher than that of the wild type (WT) [11].

Glucan phosphorylase (GP) is crucial for starch degradation and fruit quality, the phosphorolysis reaction of α-1,4-glucans is reversibly catalyzed by α-glucan phosphorylase (α-GP; EC 2.4.1.1), which produces α-D-glucose 1-phosphate (α-G1P) in chloroplasts [12]. In general, starch, glycogen, and maltodextrin serve as substrates for phosphorylation of these enzymes. The enzyme α-GP catalyzes these substrates, resulting in the production of at least 5 units of glucose residues [13]. In *Arabidopsis thaliana*, α-glucan phosphorylase (PHS1; EC 2.4.1.1) releases glucose 1-phosphate (G1P) from the glucan chains ends, followed by phosphoglucomutase form conversion of G1P to glucose 6-phosphate (G6P), giving rise to the export of G6P from the chloroplast [14]. In plants with crassulacean acid metabolism, PHS1 also plays a critical role in starch degradation in the chloroplast, and compared to the hydrolytic breakdown of starch (amylase) into maltose and glucose, this phosphorolytic degradation of starch (glucan phosphorylase) offers energy advantages [15]. Both bZIP TFs and α-GP contribute to the regulation of carbohydrate accumulation and quality formation. However, the potential link between them has not been explored.

Northwest China is the primary apple-growing region, and the soil quality in this area is relatively poor. Studies have reported that the quality of apple is strongly correlated with the application of nitrogen fertilizer [16, 17]. Therefore, it is particularly significant to explore the physiological and molecular mechanisms of nitrogen on sugar metabolism in apple fruit. However, the molecular regulatory mechanism between nitrogen and sugar metabolism has not been thoroughly characterized. In this study, transcriptome sequencing analysis revealed that MdbZIP44 responded differently to various nitrogen levels and controlled starch and glucose accumulation by modulating GP enzyme activity and *α-GP2* gene expression in apple callus and tomato fruits. Moreover, biochemical assays indicated that MdbZIP44 directly interacted with MdCPRF2-like protein, and their complexes were able to activate the expression of the *Mdα-GP2* gene. Our findings provide new insights into the molecular understanding of how MdbZIP44 regulates starch and sugar metabolism in response to nitrogen.

## Results

### Transcriptome sequencing analysis of apple fruits in different nitrogen treatments

**Figure 1:**
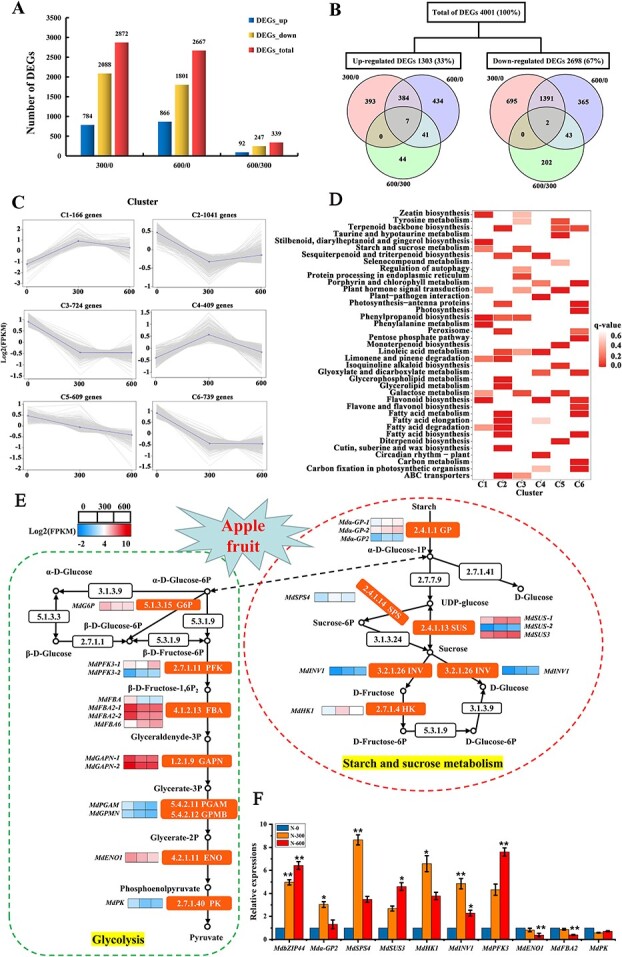
Transcriptome analysis of apple response to different nitrogen levels. (**A**) The numbers of DEGs in 300/0, 600/0, and 600/300. (**B**) Venn diagram analysis of DEGs in 300/0, 600/0, and 600/300. (**C**) Six major clusters were identified. The X-axis represents different nitrogen treatments at 60 DAFB. The Y-axis represents the value of the relative expression level (log2 (FPKM). (**D**) Functional analysis of the DEGs in 6 clusters. (**E**) Regulatory relationship of sugar metabolism pathways under different nitrogen levels. The expression of DEGs is represented by Log2 (FPKM). The color from blue to red represents the expression value from low to high. (**F**) qRT-PCR analysis of sugar metabolism-related genes. Data are expressed as means ± *SE* (Student’s *t*-test; ^*^P < 0.05, ^**^P < 0.01).

Given the physiological characteristics of apple fruits under nitrogen treatments [17], nine RNA samples were harvested from 0, 300, and 600 kg**·**hm^**−2**^ nitrogen treatments at 60 days after full bloom (DAFB) for RNA sequencing. As shown in Table S1 (see online supplementary material) the Q30 percentage exceeded 93.09%, and the GC content ranged between 47.73% and 48.02%. The mapped reads ranged from 90.76% to 91.34%, and the unique match ranged from 87.27% to 88.72%. After data compiling, 2872, 2667, and 339 genes were differently expressed at 300/0, 600/0, and 600/300, respectively. Among them, 784 genes were up-regulated and 2088 were down-regulated in 300/0, 866 were up-regulated and 1801 were down-regulated in 600/0, and 92 were up-regulated while 247 were down-regulated in 600/300 (Fig. 1A and B).

Co-expression clustering categorized 3688 differentially expressed genes (DEGs) into six clusters based on their expression characteristics (Fig. 1C). Cluster 1 consists of 166 DEGs that were rapidly up-regulated from 0 kg**·**hm^**−2**^ to 300 kg**·**hm^**−2**^, followed by a gradual down-regulation from 300 kg**·**hm^**−2**^ to 600 kg**·**hm^**−2**^. Most of these DEGs participated in pathways of ‘zeatin biosynthesis’, ‘fatty acid degradation’, ‘phenylpropanoid biosynthesis’, and ‘flavonoid biosynthesis’. Cluster 2, containing the most numerous 1041 genes, was relatively down-regulated from 0 kg**·**hm^**−2**^ to 300 kg**·**hm^**−**2^, and then gradually up-regulated from 300 kg·hm^−2^ to 600 kg**·**hm^**−2**^. This cluster is enriched in pathways of ‘photosynthesis’, ‘peroxisome’, ‘glycerolipid metabolism’, and ‘fatty acid metabolism’. Cluster 3–Cluster 6 were primarily enriched in ‘starch and sucrose metabolism’, ‘photosynthesis’, ‘carbon metabolism’, ‘fatty acid metabolism’, ‘carbon metabolism’, and ‘carbon fixation in photosynthetic organisms’ (Fig. 1D).

A total of 24 DEGs were involved in sugar metabolism, including three glycogen phosphorylase (*Mdα-GP-1*: *MD00G1156200*; *Mdα-GP-2*: *MD11G1082200*; *Mdα-GP2*: *MD05G1167500*), one sucrose-phosphate synthase (*MdSPS4*: *MD10G1002500*), three sucrose synthase (*MdSUS-1*: *MD02G1100600*; *MdSUS-2*: *MD15G1223500*; *MdSUS3*: *MD11G1307000*), one beta-fructofuranosidase (*MdINV1*: *MD12G1028200*), and one fructokinase (*MdFRK1*: *MD04G1042400*). Genes linked to glycolysis contained one glucose-6-phosphate (*MdG6P*: *MD02G1144900*), two 6-phosphofructokinase (*MdPFK3–1*: *MD08G1109700*; *MdPFK3–2*: *MD17G1180700*), four fructose-bisphosphate aldolase (*MdFBA*: *MD11G1038900*; *MdFBA2–1*: *MD00G1040000*; *MdFBA2–2*: *MD10G1063600*; *MdFBA6*: *MD12G1018700*), two glyceraldehyde-3-phosphate dehydrogenase (*MdGAPN-1*: *MD02G1009300*; *MdGAPN-2*: *MD15G1154800*), two 2,3-bisphosphoglycerate-dependent phosphoglycerate mutase (*MdPGAM: MD15G1364700*; *MdGPMB*: *MD11G1158000*), one enolase (*MdENO1*: *MD06G1208300*), and one pyruvate kinase (*MdPK*: *MD00G1031300*). The expression levels of *MdSPS4*, *MdFRK1*, *Mdα-GP*, *MdSUS*, *MdINV1*, *MdFBA6*, *MdPFK3–2* were up-regulated in 300/0 and 600/0, while *MdG6P*, *MdPGAM*, *MdENO1*, *MdPK*, *MdFBA*, and *MdFBA2* were down-regulated in 300/0 and 600/0 (Fig. 1E). Additionally, 10 DEGs closely linked to sugar metabolism were selected for the quantitative real-time-PCR (qRT-PCR) analysis to validate the data from transcriptome sequencing. These results showed that qRT-PCR expression patterns and RNA-Seq were highly consistent (Fig. 1F; Fig. S1, see online supplementary material).

### Molecular characterization of MdbZIP44

MdbZIP44 (MD10G1069900) was excavated through transcriptional sequencing analysis (Fig. 1F; Fig. S1, see online supplementary material). MdbZIP44 belongs to the S1 subfamily of apple bZIP TF. It has been reported that the S1 subfamily of plant bZIP TF is involved in sugar signal transduction [8–11]. Therefore, this paper speculates that MdbZIP44 not only responded to nitrogen, but might also be closely related to sugar metabolism. Subsequent experiments will be carried out to validate this hypothesis.

**Figure 2:**
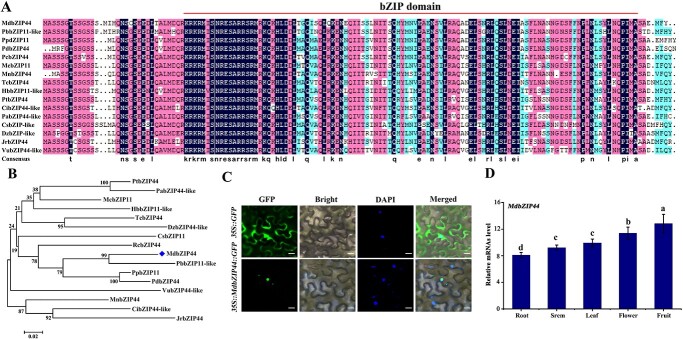
Bioinformatics analysis of MdbZIP44. (**A**) Multiple alignments of the amino acid sequences of bZIP44/bZIP11 from *Malus domestica* (Md), *Pyrus x bretschneideri* (Pb), *Prunus persica* (Pp), *Prunus dulcis* (Pd), *Rosa chinensis* (Rc), *Manihot esculenta* (Me), *Morus notabilis* (Mn), *Theobroma cacao* (Tc), *Hevea brasiliensis* (Hb), *Populus trichocarpa* (Pt), *Carya illinoinensis* (Ci), *Populus alba* (Pa), *Citrus sinensis* (Cs), *Durio zibethinus* (Dz), *Juglans regia* (Jr), and *Vigna unguiculata* (Vu). (**B**) Phylogenetic analysis of the MdbZIP44 from apple and its closest homologs. (**C**) Subcellular localization of MdbZIP44 based on GFP signal in tobacco leaves. Bars = 10 μm. (**D**) Relative mRNAs level of *MdbZIP44* in different tissues of apple.

MdbZIP44 is located on the chromosome 10 of apple genome, and its open reading frame (ORF) was 480 bp, encoding a protein of 160 amino acids. The predicted molecular weight of MdbZIP44 is ~18 kDa, with an isoelectric point of 5.93. Multiple sequence alignments revealed that bZIP44 proteins shares high sequence identity with the bZIP domain from *Malus domestica*, *Pyrus x bretschneideri*, *Prunus persica*, *Prunus dulcis*, *Rosa chinensis*, *Manihot esculenta*, *Morus notabilis*, *Theobroma cacao*, *Hevea brasiliensis*, *Populus trichocarpa*, *Carya illinoinensis*, *Populus alba*, *Citrus sinensis*, *Durio zibethinus*, *Juglans regia*, and *Vigna unguiculata* (Fig. 2A). Phylogenetic analyses showed that MdbZIP44 is highly homologous to PbbZIP11-like compared to homologous proteins of other species (Fig. 2B). For the subcellular localization of MdbZIP44, 35S: MdbZIP44-GFP or 35S: GFP were transiently transfected into tobacco leaves. The distribution of 35S: GFP fluorescence in the cell was visible through microscopic analysis, while the 35S: MdbZIP44-GFP signal was confined to the nucleus (Fig. 2C), suggesting that MdbZIP44 was a nuclear protein. Furthermore, *MdbZIP44* exhibited the highest expression level in apple fruit, followed by flower, leaf, stem, and root (Fig. 2D).

### *MdbZIP44* regulates sugar metabolism in tomato fruits and apple callus under nitrogen supply

**Figure 3:**
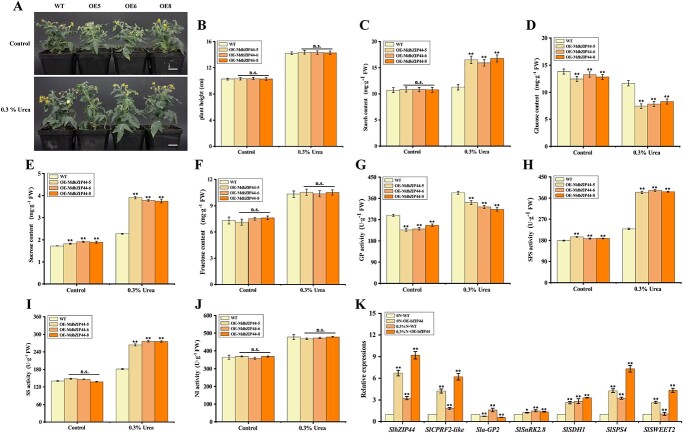
*MdbZIP44* overexpression regulates starch and sugar metabolism in tomato fruits. (**A**) The phenotypes in WT and *MdbZIP44*-overexpressing tomato plants under control and 0.3% urea treatments. Bars = 1 cm. (**B**–**J**) Plant height, starch, glucose, sucrose, and fructose content, GP, SPS, SS, and NI activities in WT and *MdbZIP44*-overexpressing tomato fruits under control and 0.3% urea treatment. (**K**) Relative expression of sugar metabolism-related genes. In (**B**–**K**) data are expressed as means ± *SE* (Student’s *t*-test; ^*^P < 0.05, ^**^P < 0.01).

*MdbZIP44* was heterologously expressed in tomato to analyse its function on sugar metabolism, resulting in the acquisition of three T3 transgenic tomato plants (OE-5, OE-6, and OE-8) (Fig. 3A; Fig. S2E, see online supplementary material). The phenotypic analysis of tomato plants revealed that 0.3% urea application resulted in an increase in plant height (Fig. 3B). Notably, the starch and sucrose content of *MdbZIP44-*overexpressing lines were significantly higher than those of wild type (WT) lines under 0.3% urea treatment. Conversely, the glucose content of *MdbZIP44-*overexpressing lines was significantly lower than those of WT lines (Fig. 3C–E). There were no discernible differences in fructose content between overexpressed and wild tomato fruits under control (no urea) and 0.3% urea treatments (Fig. 3F). In addition, GP activity of *MdbZIP44-*overexpressing lines was significantly lower than those of WT lines under the control and 0.3% urea treatments (Fig. 3G), whereas SPS activity was significantly higher than those of WT lines under the control and 0.3% urea treatments (Fig. 3H), and SS activity was significantly higher than those of WT lines only under 0.3% urea treatment (Fig. 3I). There was no significant difference in NI activity between the control and 0.3% urea treatments (Fig. 3J). Furthermore, the expression levels of *SlbZIP44* (Solyc01g109880), *SlCPRF2-like* (Solyc08g022080), *Slα-GP2* (Solyc09g031970), *SlSnRK2.8* (Solyc04g074500), *SlSDH1* (Solyc01g006510), *SlSPS4* (Solyc11g045110), and *SlSWEET2* (Solyc03g005880) were significantly higher in 0 N-OE-MdbZIP44 (without urea), 0.3%N-WT (0.3% urea), and 0.3%N-OE-MdbZIP44 than those in WT-0 N (Fig. 3K). Taken together, these findings indicated that *MdbZIP44* overexpression enhanced the accumulation of starch and sucrose in tomato fruits by regulating the activities of enzymes involved in sugar metabolism and the levels of gene expression under nitrogen treatments.

**Figure 4:**
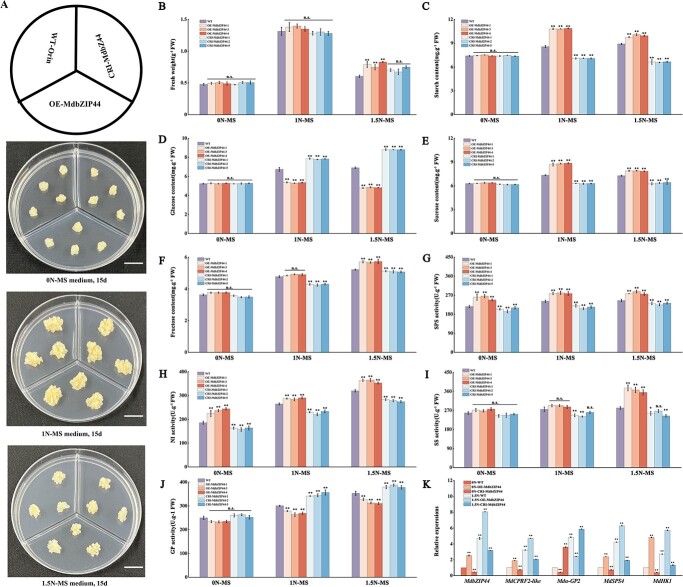
The effect of *MdbZIP44* on different nitrogen levels in transgenic apple callus. (**A**) The phenotypes of WT, OE-MdbZIP44, and CRI-MdbZIP44 transgenic apple callus were cultured on 0-MS, 1-MS, and 1.5-MS medium for 15 days. Bars = 1 cm. (**B**–**J**) Fresh weight, starch, glucose, sucrose, and fructose content, GP, SPS, SS, and NI activities in WT, OE-MdbZIP44, and CRI-MdbZIP44 transgenic apple callus under 0 N, 1 N, and 1.5 N-MS treatments. (**K**) Relative expression of sugar metabolism-related genes. In (**B**–**K**) data are expressed as means ± *SE* (Student’s *t*-test; ^*^P < 0.05, ^**^P < 0.01).

*MdbZIP44* was cloned into the relative vectors to generate *MdbZIP44-*overexpressing and silenced ‘Orin’ callus lines (Fig. S2A–D, see online supplementary material). Subsequently, WT, OE-MdbZIP44, and CRI-MdbZIP44 apple callus were treated with varying concentrations of nitrogen (0 N, 1 N, and 1.5 N-MS). Phenotype and fresh weight results showed that apple callus grew better on 1 N-MS medium than 0 N, and 1.5 N-MS (Fig. 4A and B). The starch, sucrose, and fructose content, SPS, and NI activities of *MdbZIP44*-overexpressing callus lines in 1 N- and 1.5 N-MS medium were significantly higher than those of WT lines compared to 0 N-MS medium, whereas *MdbZIP44* silenced callus lines showed significantly lower levels than those of WT lines. Meanwhile, glucose content and GP activity of OE-MdbZIP44 and CRI-MdbZIP44 apple callus exhibited an opposite trend to starch content compared to WT lines (Fig. 4C–J). In addition, qRT-PCR showed that the expression levels of *MdbZIP44*, *MdCPRF2-like*, *Mdα-GP2*, *MdSPS4*, and *MdHK1* in 0 N-OE-MdbZIP44, 1.5 N-WT, and 1.5 N-OE-MdbZIP44 were higher than those in 0 N-WT. In contrast, the expression levels of those in 0 N-CRI-MdbZIP44, and 1.5 N-CRI-MdbZIP44 were lower than those in 0 N-WT (Fig. 4K), and the results indicated that nitrogen could activate the expression levels of these genes.

### MdbZIP44 physically interacts with MdCPRF2-like

**Figure 5:**
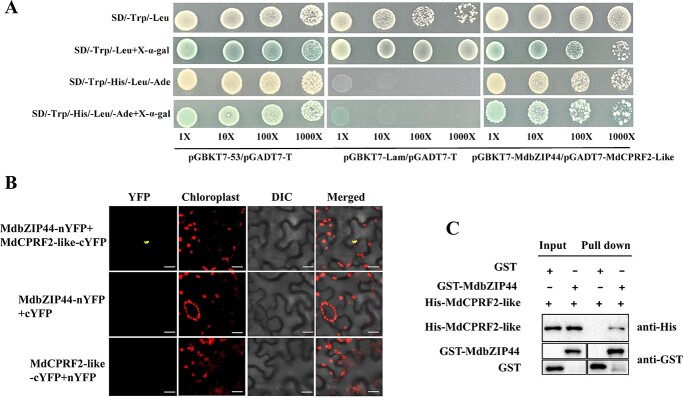
MdbZIP44 physically interacts with MdCPRF2-like. (**A**) MdbZIP44 interacted with MdCPRF2-like in a Y2H assay. (**B**) BiFC assay showing the interaction between MdbZIP44 and MdCPRF2-like. MdbZIP44-nYFP and MdCPRF2-like-cYFP interacted in the tobacco leaf cells. Scale bar = 20 μm. (**C**) Pull-down assay demonstrating the interaction between MdbZIP44 and MdCPRF2-like. MdbZIP44-GST was expressed in *Escherichia coli*, resulting in the precipitation of MdCPRF2-like-HIS using anti-GST and anti-HIS antibodies, respectively. GST alone was used as the control.

Using MdbZIP44 as bait, potential MdbZIP44-interacting proteins were screened via a yeast two-hybrid (Y2H) cDNA library. MdCPRF2-like (MD05G1206400) was selected as a candidate for interaction with MdbZIP44. pGBKT7-MdbZIP44 and pGADT7-MdCPRF2-like were co-transformed into Y2Hold yeast capable cells, and simultaneously transferred into pGBKT7–53/pGADT7-T and PGBKT7-Lam/pGADT7-T as positive and negative controls for the experiment, which were spread on the petri plates containing different components. The yeast cells transfected with pGBKT7-MdbZIP44 and pGADT7-MdCPRF2-like recombinant plasmid grew blue strain on the four-deficient plate, while the yeast cells not transfected with pGBKT7-MdbZIP44 and pGADT7-MdCPRF2-like recombinant plasmid did not grow on the four-deficient plate, but grew on the two-deficient plate, and only showed white strain (Fig. 5A). Therefore, these results suggested that MdbZIP44 interacts with MdCPRF2-like *in vitro*. Additionally, a GST pull-down assay demonstrated an *in vitro* physical interaction between His-tagged MdCPRF2-like and GST-tagged MdbZIP44 (Fig. 5C)*.*

The *in vivo* bimolecular fluorescence complementation (BiFC) assay was carried out to further validate the relationship between MdbZIP44 and MdCPRF2-like. To create the constructs MdbZIP44-nYFP and MdCPRF2-like-cYFP, MdbZIP44 and MdCPRF2-like were ligated to the N- and C-termini of YFP, respectively. Then, the vectors containing different plasmids were transferred into tobacco leaves in different combinations using an *Agrobacterium*-mediated method. The results indicated that the combination of MdbZIP44-nYFP and MdCPRF2-like-cYFP constructs showed a strong YFP signal. However, the two other combinations (MdbZIP44-nYFP + cYFP and nYFP + MdCPRF2-like-cYFP) utilized as negative controls did not show any signal (Fig. 5B). These findings demonstrated an *in vivo* physical interaction between MdbZIP44 and the MdCPRF2-like protein. Additionally, microscopic observation demonstrated that the 35S: GFP signal was observed throughout the tobacco leaf cell, whereas the fluorescence of 35S: MdCPRF2-like-GFP fusion protein was only targeted to the nucleus, suggesting that MdCPRF2-like was a nuclear protein (Fig. S3, see online supplementary material).

### MdbZIP44 binds to the promoter of *Mdα-GP2* and inhibits its transcription

**Figure 6:**
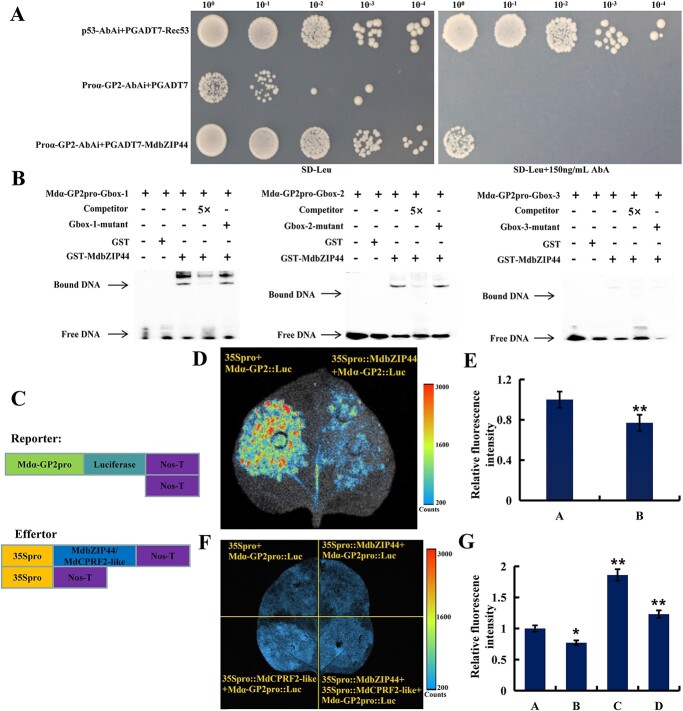
MdbZIP44 binds to the *Ma-GP2* promoter and suppresses its expression. (**A**) MdbZIP44 binds to *Mdα-GP2* promoter in yeast one-hybrid assay. The AbA screening concentration was 150 ng**/**mL. Positive controls were P53-AbAi + PGADT7-Rec53. Negative controls were Proα-GP2-AbAi + PGADT7. (**B**) EMSA assays were applied to identify the interaction between MdbZIP44 and labeled DNA probes in the *Mdα-GP2* promoter. (**C**) Luc reporter vector (Mdα-GP2pro::Luc) and effector vector (35Spro::MdbZIP44). (**D**) MdbZIP44 suppresses Mdα-GP2pro::Luc expression in tobacco leaves. (**E**) Luminescence intensity of 35Spro + Mda-GP2pro::Luc (**A**) and 35Spro::MdbZIP44 + Mdα-GP2pro::Luc (**B**). Data are expressed as means ± *SE* (Student’s *t*-test; ^*^P < 0.05, ^**^P < 0.01). (**F**) MdbZIP44 suppresses Mdα-GP2pro::Luc expression in tobacco leaves. MdCPRF2-like promotes Mdα-GP2pro::Luc expression in tobacco leaves. MdbZIP44 + MdCPRF2-like promotes Mdα-GP2pro::Luc expression in tobacco leaves. (**G**) The luminescence intensity of 35Spro + Mda-GP2pro::Luc (**A**), 35Spro::MdbZIP44 + Mdα-GP2pro::Luc (**B**), 35Spro::MdCPRF2-like + Mdα-GP2pro::Luc (**C**), and 35Spro::MdbZIP44 + 35Spro::MdCPRF2-like+Mdα-GP2pro::Luc (**D**). Data are expressed as means ± *SE* (Student’s *t*-test; ^*^P < 0.05, ^**^P < 0.01).

bZIP proteins can recognize the G-box (5′-CACGTG-3′) *cis*-element in the promoter region of downstream target genes. In this study, we identified three G-box *cis*-elements present in the *Mdα-GP2* promoter. The 2000 bp *Mdα-GP2* promoter was fused to the pAbAi vector, and MdbZIP44 was attached to the activation domain PGADT7, respectively. When the fused Mdα-GP2pro::pAbAi was co-expressed with MdbZIP44-AD, the transformant yeast cells showed adequate growth on SD/−Leu/AbA +150 ng/mL plates. However, the growth was absent in the negative control, in which both Mdα-GP2pro::pAbAi were co-expressed with the AD empty vector (Fig. 6A). These findings were further confirmed by the yeast one-hybrid (Y1H) assay, which demonstrated that MdbZIP44 specifically binds to the *Mdα-GP2* promoter.

Alternatively, the regulatory relationship between MdbZIP44 and *Mdα-GP2* was also verified using electrophoretic mobility shift assay (EMSA). The experiments were performed using purified recombinant His-MdbZIP44 fusion proteins and DNA fragments containing G-box sequences from the *Mdα-GP2* promoter region as probes. The results showed that when the G-box sequence was utilized as a labelled probe, unique DNA-MdbZIP44 protein complexes could be identified. However, when additional unlabeled G-box competition probes with the same sequence were added, the formation of these complexes was significantly reduced, while this competition was not present in the mutant probe *Mdα-GP2m* (Fig. 6B). This particular competition proved that a G-box recognition sequence is necessary for the MdbZIP44 protein to bind with DNA sequences. These findings illustrated that MdbZIP44 specifically binds to G-box *cis*-elements within the *Mdα-GP2* promoter.

To verify whether MdbZIP44, MdCPRF2-like, and MdbZIP44 + MdCPRF2-like activate or suppress the expression of *Mdα-GP2*, a construct with *Mdα-GP2* fused to the reporter gene luciferase (Mdα-GP2pro::Luc) was combined with the 35Spro::MdbZIP44, 35Spro::MdCPRF2-like, and 35Spro::MdbZIP44 + 35Spro::MdCPRF2-like construct for co-infiltration into tobacco leaves (Fig. 6C). The co-expression of 35Spro::MdbZIP44 + Mdα-GP2pro::Luc, 35Spro::MdCPRF2-like + Mdα-GP2pro::Luc, and 35Spro::MdbZIP44 + 35Spro::MdCPRF2-like + Mdα-GP2pro::Luc constructs exhibited extensively lower, higher, and higher luminescence signals than the controls, respectively (Fig. 6D–G). These results support the conclusion that MdbZIP44 suppresses *Mdα-GP2* expression, MdCPRF2-like promotes *Mdα-GP2* expression, and MdbZIP44 in complex with MdCPRF2-like promotes *Mdα-GP2* expression by regulating *Mdα-GP2* transcription *in vivo*, respectively.

### MdbZIP44 promotes starch accumulation and decreases glucose content by inhibiting *Mdα-GP2* expression in apple fruits

**Figure 7:**
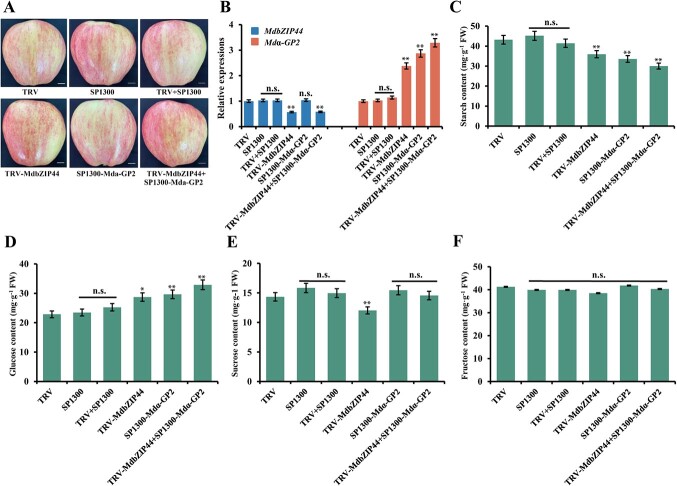
MdbZIP44 modulates sugar metabolism by regulating *Mdα-GP2* expression in apple fruits. (**A**) *MdbZIP44* and *Mdα-GP2* transient expression in vectors. *MdbZIP44* antisense cDNA fragment was ligated into the TRV vector. *Mdα-GP2* fragment was ligated into the SP1300 vector. Controls were empty vectors. Bars = 1 cm. (**B**) Relative expression of *MdbZIP44* and *Mdα-GP2* at injection sites. (**C**–**F**) Starch, glucose, sucrose, and fructose content in injected apple fruit. In (**B**–**F**) data are expressed as means ± *SE* (Student’s *t*-test; ^*^P < 0.05, ^**^P < 0.01).

A viral vector-based method (vector SP1300 for overexpression and vector TRV for suppression) was used to investigate whether MdbZIP44 regulates starch and glucose accumulation by influencing *Mdα-GP2* expression in apple fruits. TRV–MdbZIP44, SP1300–Mdα-GP2, and TRV–MdbZIP44 + SP1300–Mdα-GP2 constructs were produced, and the empty vectors were used as controls (TRV, SP1300, and TRV + SP1300 vectors) (Fig. 7A). The results showed that the *MdbZIP44* expression was decreased in TRV–MdbZIP44 apple fruits, while *Mdα-GP2* expression was increased in SP1300–Mdα-GP2 apple fruits compared to controls (Fig. 7B), qRT-PCR results showed that the transient apple fruits system was successful. Notably, when *MdbZIP44* was inhibited and *Mdα-GP2* expression was activated, the expression level of *Mdα-GP2* gene was affected by *MdbZIP44* (TRV–MdbZIP44 + SP1300–Mdα-GP2 compared to SP1300–Mdα-GP2)*,* whereas *MdbZIP44* gene expression was not affected by *Mdα-GP2* (TRV–MdbZIP44 + SP1300–Mdα-GP2 compared to TRV–MdbZIP44). This suggested that *MdbZIP44* further regulates sugar metabolism in apple fruits by regulating the expression of the *Mdα-GP2* gene. In addition, either *MdbZIP44* suppression or *Mdα-GP2* overexpression alone decreased starch content and increased the glucose content in apple fruits compared with the controls. Meanwhile, the co-infiltration of TRV–MdbZIP44 and SP1300–Mdα-GP2 decreased starch content and increased glucose content to a greater extend compared with the controls (Fig. 7C and D). It is worth noting that the suppression of *MdbZIP44* decreased sucrose content (Fig. 7E). Moreover, there were no noticeable differences in fructose content when TRV–MdbZIP44 or SP1300–Mdα-GP2 was injected alone or co-injected with TRV–MdbZIP44 and SP1300–Mdα-GP2 compared with the controls (Fig. 7F).

## Discussion

### DEGs associated with sugar metabolism pathway under different nitrogen levels

Sugar metabolism is known to be associated with plant growth, development, and stress tolerance [18, 19]. Genes related to the sugar metabolism pathway have been extensively studied in many plants [20–22]. In this study, a total of 24 genes were identified as DEGs relevant to starch and sugar metabolism under different nitrogen levels. Glycolysis generates energy for cell metabolism and helps cells adapt to abiotic stresses, such as drought, cold, and salt [23]. Phosphofructokinase (PFK) (EC 2.7.1.11), the main rate-limiting enzyme in the glycolysis pathway, catalyzes the irreversible phosphorylation of D-fructose-6-phosphate (F-6-P) to D-fructose-1,6-bisphophate (F-1,6-BP) [23]. PFKs are found in plastids and cytosol in many plants, such as potato [24], tomato [25], and ricinus [26]. In the present study, *MdFPK3* (MD08G1109700) was up-regulated under different nitrogen levels compared with the control, suggesting that *MdFPK3* contributes to accelerating the sugar degradation helping plants better adapt to multiple stress responses.

Sucrose can be converted into glucose and fructose by invertases (INV: EC 3.2.1.26) and sucrose synthases (SuSy: EC 2.4.1.13) when it enters the vacuole [27]. Studies have shown that INV enzyme plays an important role in glucose accumulation in fava bean [28], sucrose/hexose ratio in longan [29], and endosperm nuclear division in cotton [30]. In the present study, *MdINV1* (MD12G1028200) expression was up-regulated under different nitrogen levels compared to the control. This suggests that *MdINV1* may play a positive regulatory role in glucose or fructose accumulation in apple. SPS is a crucial enzyme for the production of sucrose from uridine diphosphate (UDP)-glucose and fructose-6-phosphate (SPS: EC 2.4.1.14). The research showed that *SPS* gene overexpression can significantly improve plant growth and sucrose accumulation in *Arabidopsis* [31]*,* tobacco [32], strawberry [18], pumpkins [33], sugarcane [34], and peach [21]. Furthermore, previous research has shown the up-regulation of *MdSPS5* and *MdSPS6* expressions, which can promote sucrose accumulation in apple [22]. In this study, the expression of *MdSPS4* (MD10G1002500) was up-regulated under different nitrogen levels compared with the control. The results indicated that *MdSPS4* can promote sucrose accumulation at the early fruit development stage in apple, which could be consistent with those of a previous study [22]. Several TFs are involved in plant sugar metabolism and regulate the expression of downstream target genes [35, 36]. We hypothesize that these TFs may regulate the sugar metabolism in apple fruits by regulating the expression of the DEGs. However, the mechanism of this transcriptional regulation requires further investigation.

### MdbZIP44 interacts with MdCPRF2-like and regulates the expression of *Mdα-GP2* gene

Multiple bZIP TFs are associated with sugar signaling in *Arabidopsis*, including bZIP1, 2, 11, 44, and 53 from the S1 subgroup, and bZIP9, 10, 25, and 63 from the C subgroup [7, 36]. Two orthologs of AtbZIP11 were found in tomato, named SlbZIP1 and SlbZIP2. When these orthologs were overexpressed in tomato fruits, a 1.5-fold increase in sugar content (sucrose, glucose, and fructose) was observed in tomato fruits compared to WT tomato fruits [8]. In the present study, the apple bZIP TF from transcriptome is known as MdbZIP44 in the apple database and MdbZIP11 in the National Center for Biotechnology Information (NCBI). Multiple sequence alignments showed that MdbZIP44 had the highest homology with the bZIP11/bZIP44 genes in other species (Fig. 2A). This study suggested that apple bZIP11/bZIP44 belongs to the S1 subgroup within the apple bZIP family and speculated that it was closely related to sugar metabolism. Moreover, the late-stage experiments were conducted to verify this hypothesis.

bZIP subfamily members may interact with non-bZIP proteins to form specific regulatory complexes in the nucleus or cytoplasm [21]. SnRK1 is an essential regulator of nitrogen and carbon metabolism in plants. Research reported that PpSnRK1α interacted with PpbZIP11 to improve trehalase activity. Moreover, *PpSnRK1α* overexpression enhanced *PpbZIP11* transcriptional activity and regulated trehalose metabolism for the purpose of protecting plants from external stresses [37]. In addition, various subfamily genes of the bZIP family often interact to form homologous or heterologous dimers in the form of α-helices, and bind to specific gene promoters through their basic regions, thereby regulating the expression of downstream genes associated with relevant biological processes [38]. MdCPRF2-like protein is a member of the bZIP family in apple, and *MdbZIP44* overexpression can improve the expression of *MdCPRF2-like* in transgenic apple callus (Fig. 4K). Subcellular localization showed that both MdbZIP44 and MdCPRF2-like were located in the nucleus, and the interaction between MdbZIP44 and MdCPRF2-like was further confirmed *in vivo* and *in vitro*. Furthermore, α-GP catalyzes starch with a minimum chain length of five glucose residues [13]. Yeast one-hybrid, EMSA, and dual-luciferase assays have demonstrated that MdbZIP44 not only bound to the G-box element of the *Mdα-GP2* gene promoter, but also transcriptionally inhibited the expression of *Mdα-GP2* gene. Interestingly, MdCPRF2-like protein, the combination of MdbZIP44 and MdCPRF2-like proteins activated the expression of the *Mdα-GP2* gene (Fig. 6F and G). In summary, our findings indicate that MdbZIP44 protein and its interaction protein MdCPRF2-like have opposite effects on the regulation of downstream target genes, which may contribute to maintaining the balance of starch and sugar metabolism in apple fruits.

### MdbZIP44 regulates starch and sugar metabolism under nitrogen treatments

Nitrogen is the primary mineral nutrient element, which can affect respiration rate, photosynthesis, carbohydrate, and sugar metabolism in plants by synthesizing active substances such as nucleic acids, proteins, hormones, vitamins, and several enzymes involved in sugar metabolism in plant cells [39]. The growth and development of plants largely rely on the balance between C and N metabolism, with N metabolism closely linked to C metabolism [40–42]. In this study, *MdbZIP44*-overexpressing tomato plants treated with 0.3% urea resulted in taller plants compared to the control (no urea) (Fig. 3B), suggesting that nitrogen promoted the tomato plants growth under certain conditions. Conversely, when *MdbZIP44-*overexpressing apple callus were treated with different nitrogen levels, the growth of apple callus in 1.5 N-MS medium were weaker than that in 1 N-MS medium (Fig. 4B), indicating that excessive nitrogen can inhibit the growth of apple callus. Overall, within a suitable range, nitrogen positively regulates plant growth.

Nitrogen levels affected the synthesis, transport, and metabolism of carbohydrates in crabapple, and the expression of *MdA6PR* and *MdSPS* were up-regulated under high nitrogen levels, leading to sorbitol and sucrose synthesis in the stem tips [43]. In the present study, *MdbZIP44*-overexpressing tomato fruits and apple callus were treated with nitrogen and observed that the GP activity and the expression of α*-GP2* in *MdbZIP44*-overexpressing tomato fruits and apple callus were significantly lower than those in WT lines (Figs 3G and K, and 4G and K), suggesting that *MdbZIP44* could respond to nitrogen and inhibit GP enzyme activity and *α-GP2* gene expression. Meanwhile, *MdbZIP44*-overexpressing tomato fruits and apple callus had higher starch content and lower glucose content compared to WT lines (Figs 3C and D, and Fig. 4C and D). It is speculated that *MdbZIP44* overexpression increased starch content and decreased glucose content by negatively regulating GP enzyme activity and *α-GP2* gene expression. In addition, sucrose content, SPS, and SS activities, and the expression of sugar metabolism-related genes in *MdbZIP44*-overexpressing tomato fruits and apple callus were significantly higher than those in WT lines. It is suggested that *MdbZIP44* can promote sucrose accumulation by regulating the activities of sucrose-related enzymes and the expression of sugar metabolism-related genes. Overall, *MdbZIP44* is a crucial regulator associated with sugar and nitrogen metabolism.

## Conclusions

**Figure 8:**
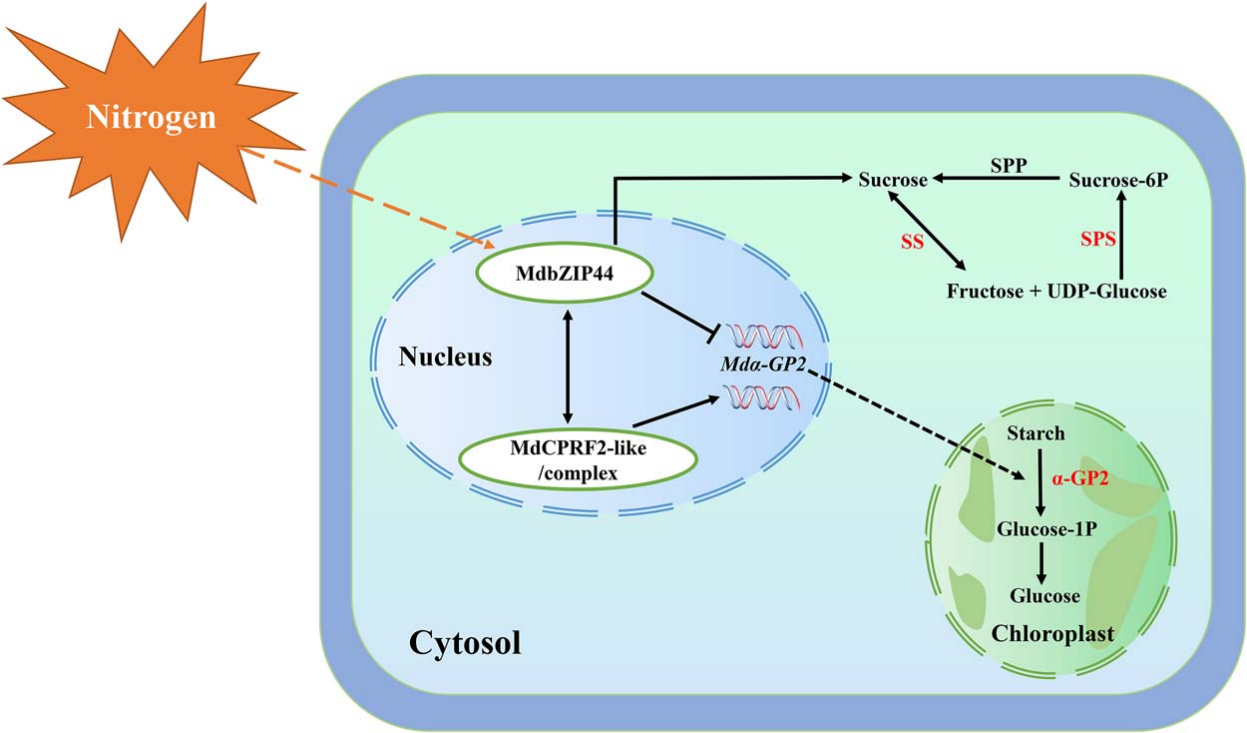
Working model in which MdbZIP44 interacts with MdCPRF2-like to regulate starch and sugar metabolism by modulating Mdα-GP2 gene expression under nitrogen supply. Different nitrogen levels rapidly induce the transcription of MdbZIP44 and MdbZIP44 modulates starch accumulation by directly suppressing Mdα-GP2 expression. In addition, MdbZIP44 interacting protein MdCPRF2-like and their complex promote Mdα-GP2 expression by regulating Mdα-GP2 transcription. The key enzymes discussed in this study are highlighted in red color. SPP, sucrose phosphate phosphatase; SPS, sucrose phosphate synthase; SS, sucrose synthase.

*MdbZIP44* was identified from transcriptome at the young fruit stage in apple*,* and this study proposed a working model for MdbZIP44 interacting with MdCPRF2-like to regulate starch and sugar metabolism by modulating *Mdα-GP2* gene expression under nitrogen supply (Fig. 8). Nitrogen rapidly induced the transcription level of *MdbZIP44*, then MdbZIP44 suppressed *Mdα-GP2* expression, thereby accumulating more starch content and less glucose content. On the other hand, MdCPRF2-like, as well as the complex of MdbZIP44 and MdCPRF2-like proteins, could activate the expression of *Mdα-GP2*. In addition, *MdbZIP44* could promote sucrose accumulation by conferring the activities of sucrose metabolism-related enzymes and the expression of sugar metabolism-related genes in apple. Overall, this study provided a new perspective for revealing the mechanism of how MdbZIP44 interacts with MdCPRF2-like to regulate starch and sugar metabolism by modulating *Mdα-GP2* gene expression under nitrogen supply.

## Materials and methods

### Plant materials and growth conditions

Fruits of apple cultivar ‘Oregon Spur Delicious’ (*M. domestica* Borkh.) were harvested at the young fruit stage in June 2020 (60 DAFB). Ten-year-old apple trees were planted in a garden of Tianshui, Gansu, China (N 39°92′, E 116°40′, 1400 m a.s.l.) at a spacing of 2 m × 4 m with the total nitrogen was 1.25 g/kg, total phosphorus was 1.42 g/kg, and total potassium was 39.38 g/kg.

In this experiment, urea (the purity is about 99%, and the nitrogen content is about 46.65%) was used as nitrogen fertilizer and there were three different nitrogen fertilizer treatments: 0, 300, and 600 kg·hm^−2^. On this basis, the fertilization was divided into three stages: bud break stage (20 April 2020), fruit expansion stage (18 June 2020), and one week before fruit ripening (20 August 2020), contributing 50%, 30%, and 20% of the total nitrogen applied in turn. The fertilizer was applied in-hole fertilization, digging four holes with a diameter of 20 cm and a depth of about 30 cm in the tree tray in the shape of plum blossoms. Fertilizer was applied into the holes and mixed thoroughly with the soil. Three comparable-growth fruit trees were selected as replicates for each treatment, which consisted of nine trees.

‘Orin’ apple callus was subcultured once at 3-week intervals on Murashige and Skoog (MS) medium plus 0.4 mg/L 6-BA and 1.5 mg/L 2,4-dichlorophenoxyacetic acid (2,4-D) at 25°C in the dark. Then, the MdbZIP44-overexpressing and WT apple callus were cultured under the same conditions in 0 N-, 1 N-, and 1.5 N-MS medium (KNO_3_ and NH_4_NO_3_ in MS medium changed, other components unchanged, 0 N-MS: no KNO_3_ and NH_4_NO_3_, 1 N-MS: normal MS medium, 1.5 N-MS: one and a half times KNO_3_ and NH_4_NO_3_) for 15-days-old.

WT and *MdbZIP44*-transformed ‘Micro-Tom’ tomato seedlings were cultivated in plastic pots (10 cm diameter) coupled with a mixture of soil, sand, and organic-mineral fertilizer (3:1:1, v:v:v) at 25 ± 2°C and 75% relative humidity for 16-h light/8-h dark. Before tomato flowering, the WT and transgenic tomato lines were treated with 0.3% urea as the experimental group, while normal conditions (no urea) served as the control group. Photographs were taken when growth differences became apparent in the tomato plants.

### RNA sequencing analysis

A total of nine different sample libraries were established for the three biological replicates of apple fruits at the young fruit stage in June 2020 (60 DAFB): 0 kg·hm^−2^, 300 kg·hm^−2^, and 600 kg·hm^−2^. The prepared libraries using apple fruits were sequenced with the Illumina HiSeq 2000 system by Novogene (Beijing, China). The *P*-value <0.01 and fold change >1.5 were set as the criteria for differentially expressed genes (DEGs) [44].

### RNA extraction, RT-PCR, and qRT-PCR

Total RNA was extracted from apple leaves using RNA Plant Plus Reagent (Tiangen, Beijing, China) and RNA was reversely transcribed into cDNA using digestion by DNase I and M-MLV reverse transcriptase (TaKaRa, Dalian, China). qRT-PCR was applied for amplification with a Light Cycler 96 instrument (Switzerland). *MdGAPDH* and *Mdactin* were used as internal controls for apple, *SlGAPDH* and *Slactin* were used as internal controls for tomato, and all the qRT-PCR figures in this study were plotted with *MdGAPDH* and *SlGAPDH* as internal controls. The gene expression values were calculated using the 2^–ΔΔCT^ method [45]. All primer sequences (Table S2, see online supplementary material) were synthesized by Sangon Biotech Co., Ltd (Shanghai, China).

### Plasmid construction and genetic transformation

To construct *MdbZIP44* and *Mdα-GP2* overexpressing or TRV vectors, the CDS sequences of *MdbZIP44* and *Mdα-GP2* were isolated from ‘Oregon Spur Delicious’ apple leaves using RT-PCR. The PCR products were linked to the pCAMBIA1300-35S-EGFP and pTRV2 vectors with the 35S promoter accordingly [46]. CRISPR/Cas9 vector is driven by the maize ubiquitin promoter (PUBI) that contains two BsaI sites and hygromycin plant selectable marker gene. Firstly, sgRNA was inserted to the region that is upstream of the sgRNA scaffold and downstream of the U3d promoter through overlapping PCR using the sgRNA-AtU3d vector. Subsequently, two different sgRNA expression cassettes were constructed in the CRISPR/Cas9 vector by using T4 ligation Golden Gate cloning and BsaI digestion [45].

Genetic transformation of ‘Orin’ apple callus and ‘Micro-Tom’ tomato plants was carried out by the *Agrobacterium*-mediated method [47].

### Soluble sugar and starch content assays

Soluble sugars were measured using an HPLC system (model 248, Waters, USA) equipped with a refractive index detector (model 2414, Waters) and an X BrigeTM amide column (3.5 mm, 4.6250 mm, USA). Briefly, 0.5 g apple pulp was extracted with 80% ethanol at 35°C for 30 min. The resulting supernatant was removed by centrifugation (13400 g, 15 min) and dried (at 60°C for 3 h). The sample was dissolved with ddH_2_O and acetonitrile then extracted using a 0.22-μm filter tip [48]. Starch assays were performed using the starch assay kit (https://www.solarbio.com/, product code: BC0700) [15].

### Assays of sugar metabolism-related enzymes

Sucrose phosphate synthase (SPS), sucrose synthase (SS), and neutral invertase (NI) were determined according to relevant assay kits (Beijing Solarbio Science & Technology Co., Ltd, product code: BC0600, BC0580, and BC0570) [27]. Glycogen phosphorylase (GP) activities were determined according to glycogen phosphorylase activity assay kit (Beijing Solarbio Science & Technology Co., Ltd, product code: BC3340) [28].

### Subcellular localization

The CDS of MdbZIP44 and MdCPRF2-like were inserted into the pCAMBIA1300 vector to generate the 35S-MdbZIP44-GFP and 35S-MdCPRF2-like-GFP expression vectors. The 35S-GFP vector was used as the control. After injecting a suspension containing recombinant plasmids into a tobacco leaf, the GFP signal was observed through a confocal laser microscope LSM880 (Zeiss, Oberkochen, Germany).

### Yeast two-hybrid assays

The MdbZIP44 CDS was inserted into the pGBKT7 vector, and the MdCPRF2-like CDS was cloned into the pGADT7 vector. The plasmids of pGBKT7-MdbZIP44 and pGADT7-MdCPRF2-like were co-transformed into Y2H Gold. The yeast strains were grown on SD/−Trp/−Leu selection medium for the transformation control and SD/−Trp/−Leu/−Ade/-His selection medium with or without X-α-gal for the interaction analysis [49].

### Pull-down assays

The MdbZIP44 CDS was connected to the pET-32a vector to create a His-tagged fusion protein. MdCPRF2-like was linked to the pGEX-4 T-1 vector to generate a GST-tagged fusion protein. The successfully connected plasmids were inserted into BL21 cells to obtain MdbZIP44-His and MdCPRF2-like-GST fusion proteins. Subsequently, a total of 0.5 mg of GST-tagged fusion protein and an equal amount of His-tagged fusion protein were combined and incubated on ice for 3 h. The resulting mixture was loaded onto columns containing glutathione sepharose 4B resin. After undergoing six washes with a wash buffer, the proteins were eluted using wash buffer supplemented with 15 mM reduced glutathione. The eluates then were separated through 12% SDS-PAGE, transferred onto PVDF membranes, and probed with anti-His antibodies, following HIS-MdbZIP44, GST-MdCPRF2-like, and empty GST proteins were pulled down [45].

### BiFC assays

The CDS of MdbZIP44 was linked to the vector 35S::pSPYNE-nYFP (MdbZIP44-nYFP) and MdCPRF2-like was linked to the vector 35S::pSPYCE-cYFP (MdCPRF2-like-cYFP). *Agrobacterium* heavy suspension carrying the recombinant plasmid (MdbZIP4-nYFP + MdCPRF2-like-cYFP, MdbZIP44-nYFP + cYFP, and nYFP + MdCPRF2-like-cYFP) were injected into tobacco leaves. The YFP fluorescence of tobacco cells was imaged with the laser confocal microscope (Zeiss LSM510) [49].

### Yeast one-hybrid assays

The full length of MdbZIP44 was ligated into the pGADT7 vector. The 2000 bp *Mdproα-GP2* were ligated into the pAbAi vector. All the fusion vectors were transferred to the Y1H Gold yeast strain, yeast cells were grown for 3 d at 28°C on SD/−Leu medium, and 150 ng/mL Aureobasidin A (AbA) was used for screening. The yeast one-hybrid assays were conducted as previously described [22].

### EMSA assays

The 5′-biotinized oligonucleotide probe was incubated with the nuclear extract at 25°C for 30 min. The entire reaction mixture was run on a non-denaturing 0.5 × TBE 6% polyacrylamide gel for 1 h at 4°C and then transferred to a Biodyne® B nylon membrane. Visualization of the signal is performed with reagents in the kit and ChemiDoc XRS (Bio-Rad Laboratories, USA) [50].

### Transient dual-luciferase assays

The promoter sequences of *Mdα-GP2* were linked to the pGreenII 0800-LUC vector (Mdα-GP2pro-LUC). MdbZIP44 was inserted into the pGreenII 62-SK vector (35Spro::MdbZIP44). Luminescence measurement was made using a living imaging apparatus (Berthold Technologies, Bad Wildbad, Germany). Firefly luciferase (LUC) and Renilla luciferase (REN) activity levels were measured as described previously [51].

### Statistical analysis

The data were analysed using one-way ANOVA (P < 0.05) or independent *t*-tests (^*^P < 0.05; ^**^P < 0.01) through IBM SPSS Statistics version 26. These values exist in the form of the mean ± standard deviation (SD) of biological triplicates.

## Acknowledgements

This research was supported by the Science and Technology Major Project of Gansu Province (22ZD6NA045), Natural Science Foundation of China (31860530), and the ‘Double-First Class’ Key Scientific Research Project of Education Department in Gansu Province (GSSYLXM-02).

## Author contributions

B.C. and J.M. designed and coordinated the research; X.C., P.W., and S.L. conducted most of the experiments and data analysis; X.C. and Z.G. jointly performed the collection, analysis, and preservation of the experimental data; X.C., Z.M., and W.L. revised the manuscript. All authors contributed to writing and editing the final manuscript.

## Data availability

All data supporting the findings are available within the paper or from the corresponding author upon request. All transcriptome sequencing data have been deposited in the NCBI Sequence Read Archive under the BioProject ID PRJNA1043230.

## Conflict of interest statement

The authors declare that they have no conflicts of interest associated with this work.

## Supplementary data

Supplementary data is available at *Horticulture Research* online.

## Supplementary Material

Web_Material_uhae072

## References

[1] Zhao TB , Dai AG. The magnitude and causes of global drought changes in the twenty-first century under a low-moderate emissions scenario. J Clim. 2015;28:4490–512

[2] Cunha MDS , Cavalcante IHL, Mancin AC. et al. Impact of humic substances and nitrogen fertilising on the fruit quality and yield of custard apple. Acta Scientiarum Agronomy. 2015;37:211–8

[3] O’Brien JA , Vega A, Bouguyon E. et al. Nitrate transport, sensing, and responses in plants. Mol Plant. 2016;9:837–56 27212387 10.1016/j.molp.2016.05.004

[4] Townsend AR , Howarth. Fixing the global nitrogen problem. Sci Am. 2010;302:64–71 20128225 10.1038/scientificamerican0210-64

[5] Wang H , Xu KX, Li XG. et al. A pear S1-bZIP transcription factor PpbZIP44 modulates carbohydrate metabolism, amino acid, and flavonoid accumulation in fruits. Hort Res. 2023;10:2662–6810 10.1093/hr/uhad140 PMC10421730 37575657

[6] Antónia G , Ste L, Said H. et al. Characterization of pollen-expressed bZIP protein interactions and the role of ATbZIP18 in the male gametophyte. Plant Reprod. 2017;30:1–17 27896439 10.1007/s00497-016-0295-5

[7] Gibalova A , Steinbachova L, Hafidh S. Characterization of pollen-expressed bZIP protein interactions and the role of ATbZIP18 in the male gametophyte. Plant Reprod. 2017;30:1–17 27896439 10.1007/s00497-016-0295-5

[8] Dietrich K , Weltmeier F, Ehlert A. et al. Heterodimers of the Arabidopsis transcription factors bZIP1 and bZIP53 reprogram amino acid metabolism during low energy stress. Plant Cell. 2011;23:381–95 21278122 10.1105/tpc.110.075390 PMC3051235

[9] Dröge-Laser W , Weiste C. The C/S1 bZIP network: a regulatory hub orchestrating plant energy homeostasis. Trends Plant Sci. 2018;23:422–33 29525129 10.1016/j.tplants.2018.02.003

[10] Sagor GHM , Berberich T, Tanaka S. et al. A novel strategy to produce sweeter tomato fruits with high sugar contents by fruit-specific expression of a single bZIP transcription factor gene. Plant Biotechnol J. 2016;14:1116–26 26402509 10.1111/pbi.12480 PMC11388862

[11] Xing SN , Chen KL, Zhu HC. et al. Fine-tuning sugar content in strawberry. Genome Biol. 2020;21:230 32883370 10.1186/s13059-020-02146-5 PMC7470447

[12] Kitaoka M , Hayashi K. Carbohydrate-processing phosphorolytic enzymes. Trends Glycosci Glycotechnol. 2002;14:35–50

[13] Cohen P . The origins of protein phosphorylation. Nat Cell Biol. 2002;4:E127–30 11988757 10.1038/ncb0502-e127

[14] Weise SE , van Wijk KJ, Sharkey TD. The role of transitory starch in C3, CAM, and C4 metabolism and opportunities for engineering leaf starch accumulation. J Exp Bot. 2011;62:3109–18 21430293 10.1093/jxb/err035

[15] Nathalie C , Johan C, Natalia HC. et al. Phosphorolytic degradation of leaf starch via plastidic α-glucan phosphorylase leads to optimized plant growth and water use efficiency over the diel phases of crassulacean acid metabolism. J Exp Bot. 2021;12:4419–34 10.1093/jxb/erab132 PMC8266541 33754643

[16] Zhang D , Yang K, Kan Z. et al. The regulatory module MdBT2–MdMYB88/MdMYB124–MdNRTs regulates nitrogen usage in apple. Plant Physiol. 2021;185:1924–42 33793944 10.1093/plphys/kiaa118 PMC8133671

[17] Cao X , Li W, Wang P. et al. New insights into MdSPS4-mediated sucrose accumulation under different nitrogen levels revealed by physiological and transcriptomic analysis. Int J Mol Sci. 2022;23:16073 36555711 10.3390/ijms232416073 PMC9782777

[18] Jia H , Wang Y, Sun M. et al. Sucrose functions as a signal involved in the regulation of strawberry fruit development and ripening. New Phytol. 2013;198:453–65 23425297 10.1111/nph.12176

[19] Li L , Sheen J. Dynamic and diverse sugar signaling. Curr Opin Plant Biol. 2016;33:116–25 27423125 10.1016/j.pbi.2016.06.018 PMC5050104

[20] Iqbal S , Ni X, Bilal MS. et al. Identification and expression profiling of sugar transporter genes during sugar accumulation at different stages of fruit development in apricot. Gene. 2020;742:144584 32173541 10.1016/j.gene.2020.144584

[21] Aslam M , Deng L, Wang X. et al. Expression patterns of genes involved in sugar metabolism and accumulation during peach fruit development and ripening. Sci Hortic. 2019;257:108633

[22] Li M , Feng F, Cheng L. Expression patterns of genes involved in sugar metabolism and accumulation during apple fruit development. PLoS One. 2012;7:e33055 22412983 10.1371/journal.pone.0033055 PMC3296772

[23] Winkler C , Delvos B, Martin W. et al. Purification, microsequencing and cloning of spinach ATP-dependent phosphofructokinase link sequence and function for the plant enzyme. FEBS J. 2007;274:429–38 17229148 10.1111/j.1742-4658.2006.05590.x

[24] Teramoto M , Koshiishi C, Ashihara H. Wound-induced respiration and pyrophosphate: fructose-6-phosphate phosphotransferase in potato tubers. Zeitschrift fur Naturforschung C. 2000;55:953–6 10.1515/znc-2000-11-1217 11204201

[25] Isaac JE , Rhodes MJC. The role of inorganic phosphate in the regulation of pfk activity in tomatoes. Phytochemistry. 1987;26:645–8

[26] Podesta FE , Plaxton WC. Regulation of cytosolic carbon metabolism in germinating Ricinus communis cotyledons. Planta. 1994;194:374–80

[27] Stein O , Granot D. An overview of sucrose synthases in plants. Front Plant Sci. 2019;10:95 30800137 10.3389/fpls.2019.00095 PMC6375876

[28] Weschke W , Panitz R, Gubatz S. et al. The role of invertases and hexose transporters in controlling sugar ratios in maternal and filial tissues of barley caryopses during early development. Plant J. 2003;33:395–411 12535352 10.1046/j.1365-313x.2003.01633.x

[29] Luo T , Shuai L, Liao LY. et al. Soluble acid invertases act as key factors influencing the sucrose/hexose ratio and sugar receding in longan (*Dimocarpus longan* Lour.) pulp. J Agric Food Chem. 2019;67:352–63 30541284 10.1021/acs.jafc.8b05243

[30] Wang L , Ruan YL. New insights into roles of cell wall invertase in early seed development revealed by comprehensive spatial and temporal expression patterns of GhCWIN1 in cotton. Plant Physiol. 2012;160:777–87 22864582 10.1104/pp.112.203893 PMC3461555

[31] Signora L , Galtier N, Skot L. et al. Overexpression of sucrose phosphate synthase in *Arabidopsis thaliana* results in increased foliar sucrose/starch ratios and favours decreased foliar carbohydrate accumulation in plants after prolonged growth with CO2 enrichment. J Exp Bot. 1998;49:669–80

[32] Seger M , Gebril S, Tabilona J. et al. Impact of concurrent overexpression of cytosolic glutamine synthetase (GS1) and sucrose phosphate synthase (SPS) on growth and development in transgenic tobacco. Planta. 2015;241:69–81 25213117 10.1007/s00425-014-2165-4

[33] Wang C , Wang Y, Wang M. et al. Soluble sugars accumulation and related gene expression during fruit development in *Cucurbita maxima* Duchesne. Sci Hortic. 2020;272:109520

[34] Anur RM , Mufithah N, Sawitri WD. et al. Overexpression of sucrose phosphate synthase enhanced sucrose content and biomass production in transgenic sugarcane. Plan Theory. 2020;9:200 10.3390/plants9020200 PMC7076389 32041093

[35] Rolland F , Baenagonzalez E, Sheen J. Sugar sensing and signaling in plants: conserved and novel mechanisms. Annu Rev Plant Biol. 2006;57:675–709 16669778 10.1146/annurev.arplant.57.032905.105441

[36] Weiste C , Pedrotti L, Selvanayagam J. et al. The Arabidopsis bZIP11 transcription factor links low-energy signalling to auxin-mediated control of primary root growth. PLoS Genet. 2017;13:e1006607 28158182 10.1371/journal.pgen.1006607 PMC5315408

[37] Zhang S , Wang H, Luo J. et al. Peach PpSnRK1α interacts with bZIP11 and maintains Trehalose balance in plants. Plant Physiol Biochem. 2021;160:377–85 33550178 10.1016/j.plaphy.2021.01.036

[38] Zulfiqar A , Sarwat SS, Karim I. et al. Functions of plant’s bZIP transcription factors. Pak J Agric Sci. 2016;53:303–14

[39] Krapp A . Plant nitrogen assimilation and its regulation: a complex puzzle with missing pieces. Curr Opin Plant Biol. 2015;25:115–22 26037390 10.1016/j.pbi.2015.05.010

[40] Palenchar PM , Kouranov A, Lejay LV. et al. Genome-wide patterns of carbon and nitrogen regulation of gene expression validate the combined carbon and nitrogen (CN)-signaling hypothesis in plants. Genome Biol. 2004;5:R91 15535867 10.1186/gb-2004-5-11-r91 PMC545782

[41] Zheng ZL . Carbon and nitrogen nutrient balance signaling in plants. Plant Signal Behav. 2009;4:584–91 19820356 10.4161/psb.4.7.8540 PMC2710548

[42] Ruffel S , Gojon A, Lejay L. Signal interactions in the regulation of root nitrate uptake. J Exp Bot. 2014;65:5509–17 25165146 10.1093/jxb/eru321

[43] Zhang L , Sun S, Liang Y. et al. Nitrogen levels regulate sugar metabolism and transport in the shoot tips of crabapple plants. Front Plant Sci. 2021;12:372 10.3389/fpls.2021.626149 PMC7988234 33777066

[44] Kim D , Pertea G, Trapnell C. et al. TopHat2: accurate alignment of transcriptomes in the presence of insertions, deletions and gene fusions. Genome Biol. 2013;14:R36 23618408 10.1186/gb-2013-14-4-r36 PMC4053844

[45] Hu DG , Yu JQ, Han PL. et al. The regulatory module MdPUB29-MdbHLH3 connects ethylene biosynthesis with fruit quality in apple. New Phytol. 2019;221:1966–82 30288754 10.1111/nph.15511

[46] Xie XB , Li S, Zhang RF. et al. The bHLH transcription factor MdbHLH3 promotes anthocyanin accumulation and fruit colouration in response to low temperature in apples. Plant Cell Environ. 2012;35:1884–97 22519753 10.1111/j.1365-3040.2012.02523.x

[47] Hu DG , Sun CH, Zhang QY. et al. Glucose sensor MdHXK1 phosphorylates and stabilizes MdbHLH3 to promote anthocyanin biosynthesis in apple. PLoS Genet. 2016;12:e1006273 27560976 10.1371/journal.pgen.1006273 PMC4999241

[48] Shao X , Zhu Y, Cao S. et al. Soluble sugar content and metabolism as related to the heat-induced chilling tolerance of loquat fruit during cold storage. Food Bioprocess Technol. 2013;6:3490–8

[49] Wang XF , An JP, Liu X. et al. The nitrate-responsive protein MdBT2 regulates anthocyanin biosynthesis by interacting with the MdMYB1 transcription factor. Plant Physiol. 2018;178:890–906 29807931 10.1104/pp.18.00244 PMC6181044

[50] Rio DC . Electrophoretic mobility shift assays for RNA-protein complexes. Cold Spring Harb Protoc. 2014;4 10.1101/pdb.prot080721 24692495

[51] An JP , Yao JF, Xu RR. et al. An apple NAC transcription factor enhances salt stress tolerance by modulating the ethylene response. Physiol Plant. 2018;164:279–89 29527680 10.1111/ppl.12724

