# Supplementary material for: MdbZIP44–MdCPRF2-like–Mdα-GP2 regulate starch and sugar metabolism in apple under nitrogen supply
Source: Hortic Res. 2024 Mar 15;11(5):uhae072. doi: 10.1093/hr/uhae072 (PMC11079487; doi:10.1093/hr/uhae072)
Supplement: Web_Material_uhae072 [file web_material_uhae072.zip › Table S1.docx]

| **DAFB (days)** | **N fertilizer** | **Clean Reads** | **Clean**  **Bases** | **GC Content** | **%≥Q30** | **Mapped Reads** | **Unique Match** |
| --- | --- | --- | --- | --- | --- | --- | --- |
| 60 | 0-1 | 22,336,116 | 6,687,143,380 | 48.08% | 93.31% | 91.23% | 88.65% |
|  | 0-2 | 24,310,920 | 7,279,223,922 | 47.88% | 93.30% | 90.65% | 88.16% |
|  | 0-3 | 20,025,264 | 5,997,767,604 | 48.09% | 94.08% | 92.15% | 89.35% |
|  | 300-1 | 20,763,401 | 6,216,574,982 | 48.14% | 92.48% | 90.78% | 87.36% |
|  | 300-2 | 21,970,467 | 6,578,538,198 | 47.92% | 93.75% | 90.87% | 86.61% |
|  | 300-3 | 21,047,464 | 6,304,436,984 | 47.58% | 93.04% | 90.62% | 87.84% |
|  | 600-1 | 21,021,399 | 6,293,937,246 | 47.85% | 93.25% | 91.18% | 87.07% |
|  | 600-2 | 19,661,729 | 5,888,843,668 | 47.64% | 93.64% | 91.16% | 88.15% |
|  | 600-3 | 21,611,645 | 6,473,908,274 | 47.71% | 93.55% | 91.40% | 87.35% |

**Table S1.** The alignment statistics result with the reference gene for all samples
