# Supplementary material for: MdbZIP44–MdCPRF2-like–Mdα-GP2 regulate starch and sugar metabolism in apple under nitrogen supply
Source: Hortic Res. 2024 Mar 15;11(5):uhae072. doi: 10.1093/hr/uhae072 (PMC11079487; doi:10.1093/hr/uhae072)
Supplement: Web_Material_uhae072 [file web_material_uhae072.zip › Table S2.docx]

**Table S2.** Primers used in this study

| **Gene** | **（5′–3′）Forward primer** | **（5′–3′）Reverse primer** |
| --- | --- | --- |
| *MdbZIP44* | ATGAGGAAGCAGAAGCACTTGGATG | GGCTTGTGATGTTGAGGCTGGAG |
| *Mdα-GP2* | ATGGTCTATTCAAGCAGCGGATCG | CACAACATCATGCCTGACAACTTCC |
| *MdSPS4* | GTGTGAAGCCAGGAGCCAAGAC | AGTGTAGACAAGGTTGCATCGGAAG |
| *MdSUS3* | CGTCTCACACTTGCCGCTCTC | TTCGTCGTCGTCGTCTTCTTCTTC |
| *MdHK1* | CCTTGCTGCTGCTGGAGTCTTG | CCACCATCCAAAGCTACCACTGTC |
| *MdINV1* | GGGAGGCAAAAGGAAGCACAGAG | GGAGCAGAGTGGAGAGGATGATGG |
| *MdPFK3* | ATGCCCGAAACACCGTTACCTTG | TTCCACGATCCTGAATGCTGTCAAC |
| *MdENO1* | TTCCACGATCCTGAATGCTGTCAAC | CACCGACTTGACCTTGTACTCCTTG |
| *MdFBA2* | GATACTCTGCTCATCCGTGCCAAG | GCCTCCTCAGACTCTCCTTCTCC |
| *MdPK* | TGAGGTATCCGACATTGCCATTGC | CAGTCCGAAGTGCGACAGTGTG |
| *Mdactin* | TTCTCGTTGAGGGCTATTCCA | CCACAGACTTCATCGGTGACA |
| *SlbZIP44* | TGGCTTCGTCAAGTGGTACATCATC | TCCTTGATCTTCTTGCCGATTCACG |
| *Slα-GP2* | GCCCACCAAGTTCCAGAATAAGACC | ACTCCCGAAGATTAGCAAGCAGTTC |
| *SlSnRK2.8* | GCAGATGTTTGGTCCTGTGGAGTC | GGAATTGAGTAGCGAGCGGTGAG |
| *SlSDH1* | AGGATGAAGGCTGTCGGTATTTGTG | GACTTCACCGCCAACTTCCTCTATG |
| *SlSPS4* | AATCCAGCCCTTGTTGAGCCATTC | GAGTAGCCCATTGTGTAGTGCCTTG |
| *SlCPRF2* | TCGTCTTCTTCACAGCAGATCAAGC | CGGAATCAATCGGAGGTTCAGAGAC |
| *SlSWEET2* | AACGGGATAGGAGGAGTTCTTGGG | CTTGTTGGCTCCTCATTAGGTCTGC |
| *Slactin* | TGTCCCTATTTACGAGGGTTATGC | AGTTAAATCACGACCAGCAAGAT |
| *MdbZIP44-*  *SP1300* | GAGCTCGGTACCCGGGGATCCATGGCTTCCTCCAGTGGGAC | GGTGTCGACTCTAGAGGATCCGTAGAACATCTCTGCAGAGGCCA |
| *MdCPRF2-like-*  *SP1300* | CGGTACCCGGGGATCCAATGCATTCAGTGTTCACCGC | CGACTCTAGAGGATCCCTACTTCTTATTCTTTGGGACTACTTCTTATTCTTTGGGACG |
| *Mdα-GP2-*  *SP1300* | gagctcggtacccggggatccATGGCACCTACTGCGAAATCG | catgtcgactctagaggatccAACTTCTTATACCGATAAACTGCACC |
| *MdbZIP44-*  *PGBKT7* | GGGGACAAGTTTGTACAAAAAAGCAGGCTTCATGGCTTCCTCCAGTGGGACATCTT | GGGGACCACTTTGTACAAGAAAGCTGGGTCTCAGTAGAACATCTCTGCAGAGGCC |
| *MdCPRF2-like-*  *PGADT7* | gtgggcatcgatacgggatcctt  ATGCATTCAGTGTTCACCGCG | cagctcgagctcgatggatcc  CTACTTCTTATTCTTTGGGACGGC |
| *MdbZIP44-*  *nYFP* | GGGGACAAGTTTGTACAAAAAAGCAGGCTTCatggcttcctccagtggga | GGGGACCACTTTGTACAAGAAAGCTGGGTCtcagtagaacatctctgcagagg |
| *MdCPRF2-like-*  *cYPF* | GGGGACAAGTTTGTACAAAAAAGCAGGCTTCATGCATTCAGTGTTCACCGC | GGGGACCACTTTGTACAAGAAAGCTGGGTCCTACTTCTTATTCTTTGGGACGGC |
| *MdbZP44-*  *GST* | AGTTCTGTTCCAGGGGCCCCTGGGATCCatggcttcctccagtgg | CGACCCGGGAATTCCGGGGATCtcagtagaacatctctgcagaggc |
| *MdCPRF2-like-*  *His* | ggctcacagagaacagattggtatgcattcagtgttcaccgcggac | cagccggatctcagtggtggtggtggtggtgctacttcttattctttggga |
| *MdbZIP44-*  *pGADT7* | cgctcatatggccatggaggccagtgaattatggcttcctccagtgggacatctt | gattcatctgcagctcgagctcgatggatctcagtagaacatctctgcagaggcc |
| *Mdα-GP2-*  *pAbAi* | aattgaaaagcttgaattcgagctcggtacctgaaagaatcgttcacataacata | ttttatatacatacagagcacatgcctcgaatacctttcaccattttcatcagtc |
| *Mdα-GP2-G1* | Aaatggacacgtgggcaggga | tccctgcccacgtgtccattt |
| *Mdα-GP2-G2* | ggattcgacgtgatcatggagt | actccatgatcacgtcgaatcc |
| *Mdα-GP2-G3* | cccacctaggtcgtgactcactt | aagtgagtcacgacctaggtggg |
| *Mdα-GP2-*  *pGreenII0800* | ttcctgcagcccgggggatccAACAGCGCGCCACT | cgctctagaactagtggatccATATTTTTTCTCTTGTTTTGTTGGAGTATTTTTTCT |
| *MdbZIP44-*  *TRV2* | agaaggcctccatggggatccttATGGCTTCCTCCAGTGGGAC | cgtgagctcggtaccggatccTCAGTAGAACATCTCTGCAGAGGC |
