# Supplementary figures and images for: MdbZIP44–MdCPRF2-like–Mdα-GP2 regulate starch and sugar metabolism in apple under nitrogen supply
Source: Hortic Res. 2024 Mar 15;11(5):uhae072. doi: 10.1093/hr/uhae072 (PMC11079487; doi:10.1093/hr/uhae072)

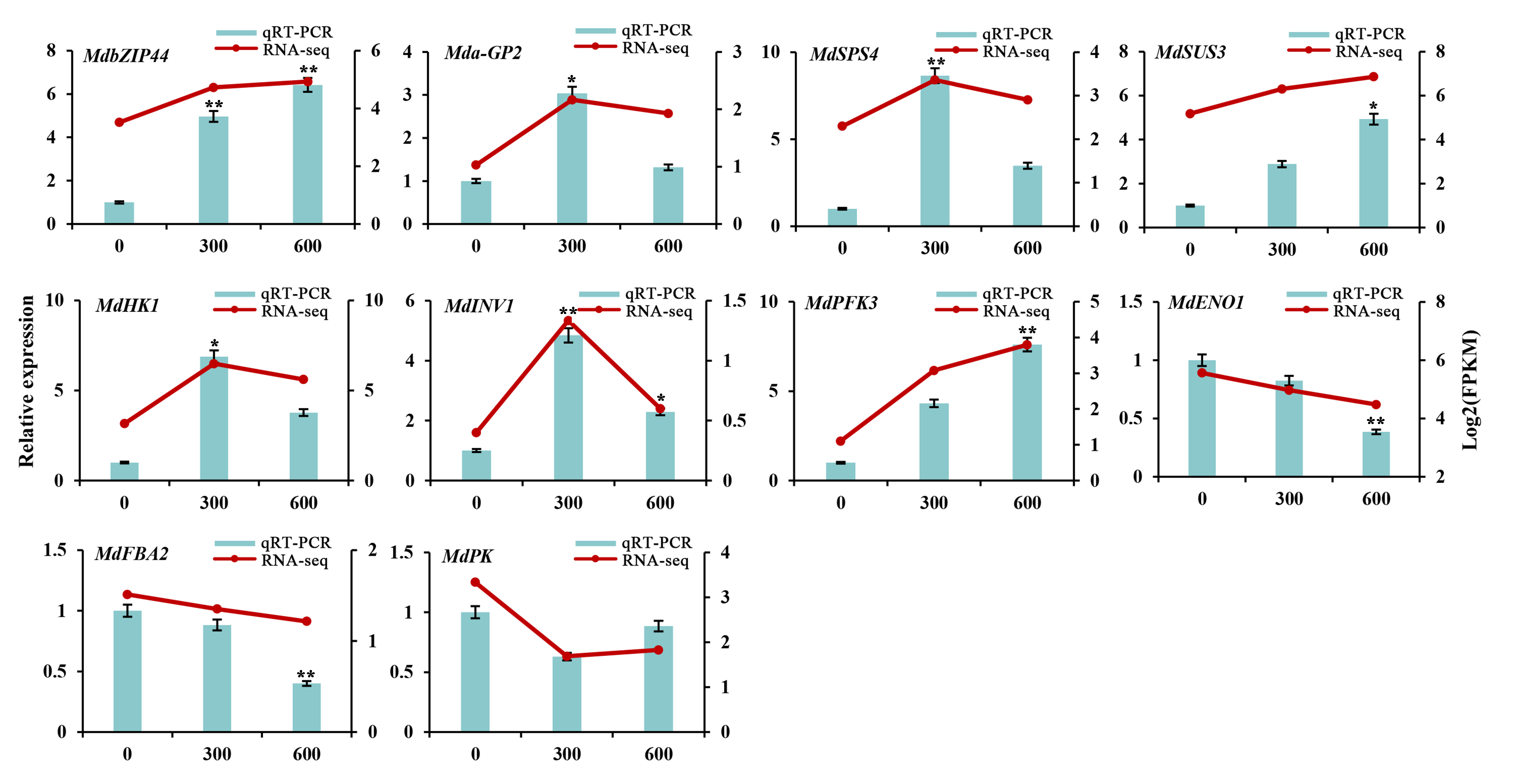

Supplement: Web_Material_uhae072 [file web_material_uhae072.zip › Fig.S1.png]

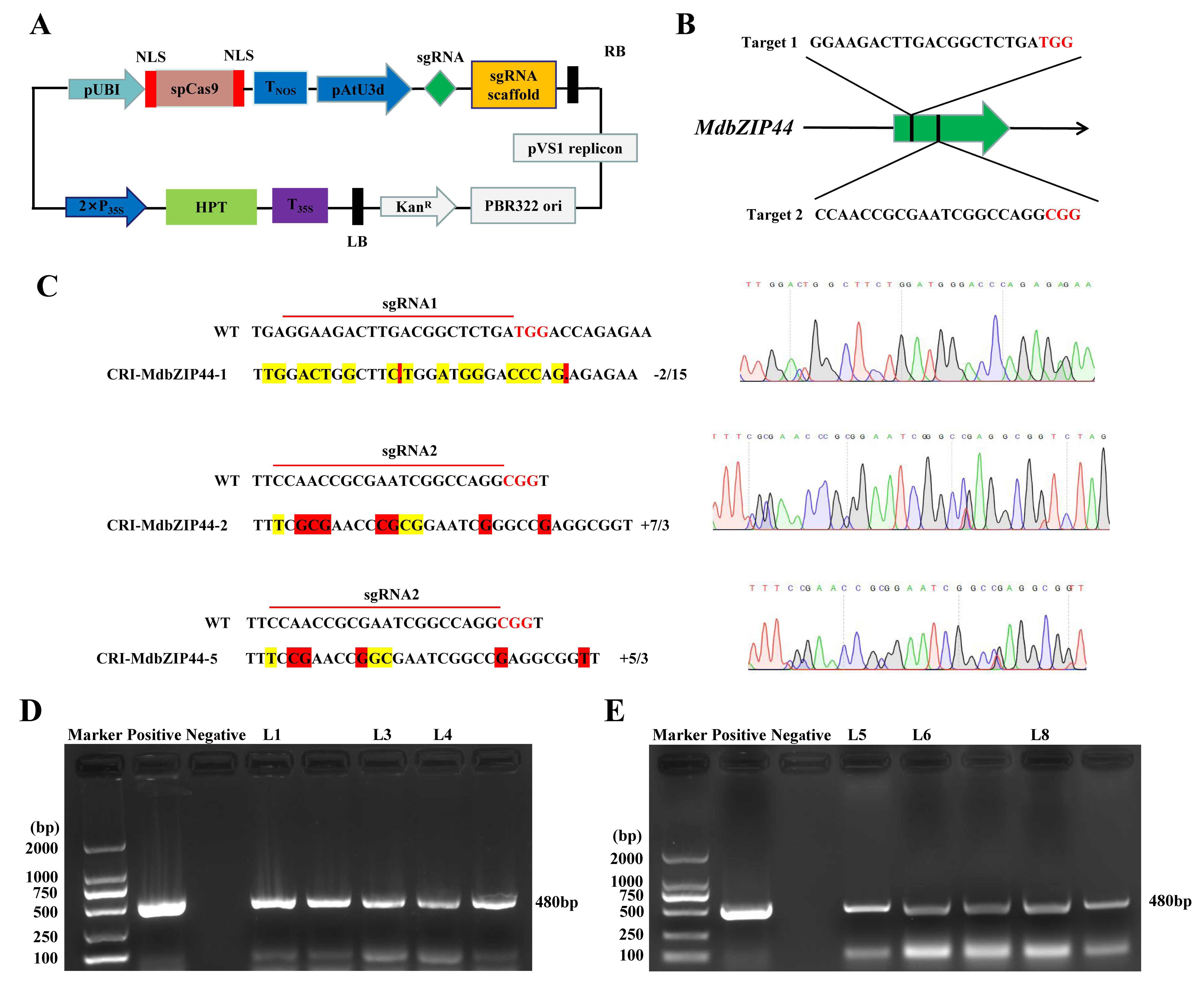

Supplement: Web_Material_uhae072 [file web_material_uhae072.zip › Fig.S2.jpg]

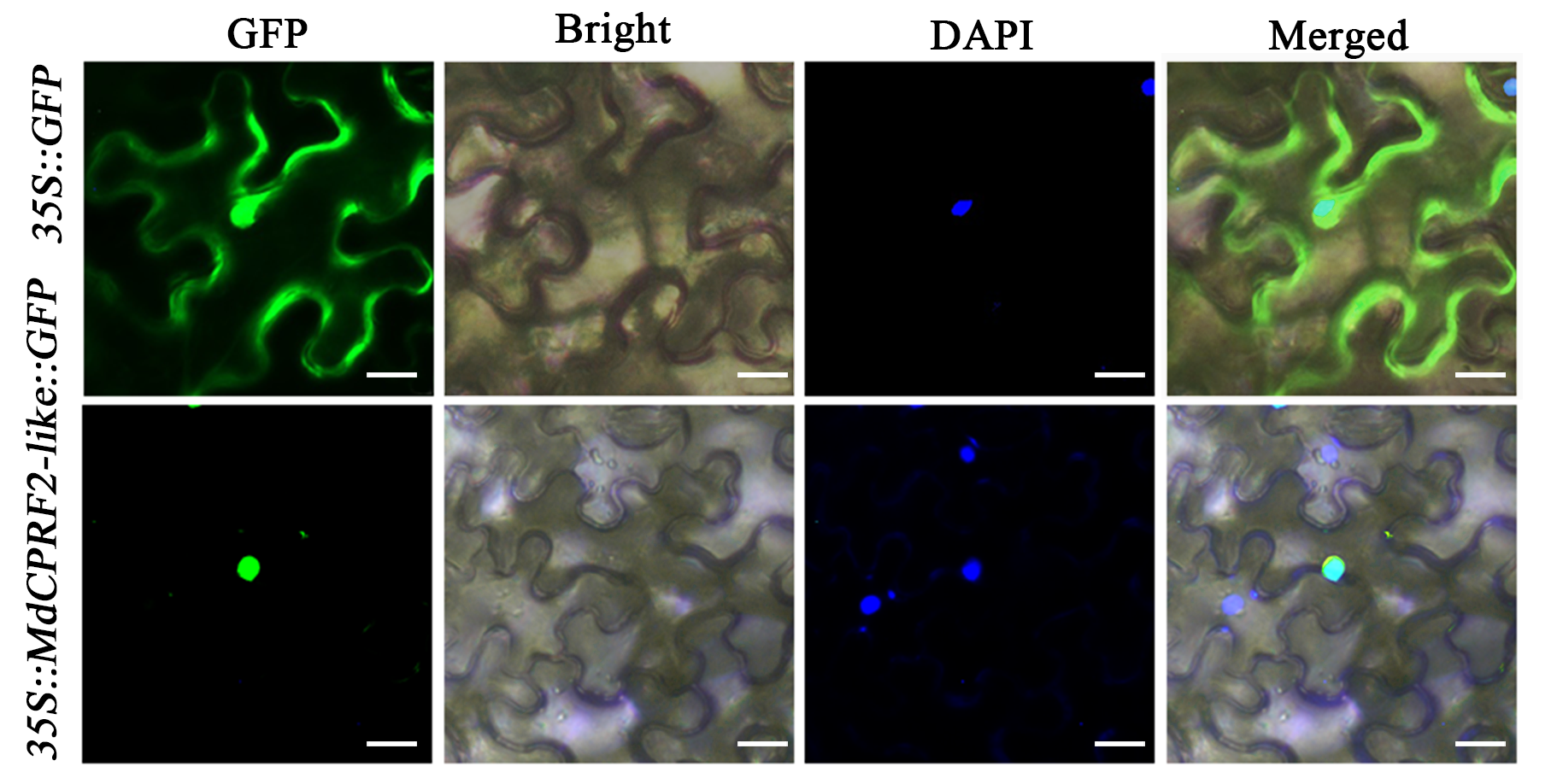

Supplement: Web_Material_uhae072 [file web_material_uhae072.zip › Fig.S3.png]
